# The importance of abilities in inclusive experiences from the perspective of people with visual impairments: the example of alpine skiing

**DOI:** 10.3389/fspor.2025.1587603

**Published:** 2025-05-30

**Authors:** Felix Oldörp, Theresa Schaller, Michelle Grenier, Martin Giese

**Affiliations:** ^1^ Faculty of Natural and Social Sciences, Sports Science & Sports Education, Heidelberg University of Education, Heidelberg, Germany; ^2^ Department of Kinesiology, University of New Hampshire, Durham, NH, United States; ^3^ Department of Education, Sport Pedagogy, Philipps-Universität Marburg, Marburg, Germany

**Keywords:** ableism, disability sport, inclusion, para-skiing, visual impairment and blindness

## Abstract

**Background:**

Alpine skiing offers both opportunities and challenges for individuals with visual impairments. Despite its potential for inclusion, barriers persist that limit participation. Using an ableism-critical and interpretivist perspective, this study explores inclusion as a subjective experience from the perspective of alpine skiers with visual impairments, focusing on how sport-related abilities influence inclusive processes. The aim is to derive implications for the development of more inclusive sports practices.

**Methods:**

A qualitative approach was used, drawing on episodic interviews with six adult skiers (five women, one man) with visual impairment from Germany, including both recreational to competitive athletes. A qualitative content analysis identified key themes related to skiing, abilities, and inclusion.

**Results:**

A synthesis of the interview results revealed four key themes: (1) skiing as a booster for self-confidence, (2) skiing as proof of expertise and competence, (3) skiing under the radar, (4) skiing as a pathway to accessibility and inclusion. Participants reported increased self-confidence, improved motor and social skills, and enhanced advocacy for accessibility. While skiing itself was seen as inclusive, structural barriers including a lack of guides, a limited awareness of para-skiing, and segregated competitions restricted full participation. Conversations with sighted individuals revealed admiration for skiers’ abilities while but also exposed gaps in their understanding of adaptive skiing.

**Conclusions:**

Skiing fosters independence, competence, and inclusion for individuals with visual impairment by enhancing skills and challenging ableist perceptions. However, systemic barriers, limited coverage and separate competitive structures, hinder full inclusion—as defined by the United Nations Convention on the Rights of Persons with Disabilities (UN CRPD)—and influence the subjective feeling of inclusion. Addressing these challenges requires greater awareness, improved access to guiding and policy changes to ensure equal participation. This study contributes to the discussion on ability, ableism, and the role of sports in inclusive practices.

## 1 Introduction

The United Nations (1), the International Paralympic Committee (2) or Special Olympics (3) see sport as a means of successfully including individuals with disabilities. Abilities are often seen as a decisive characteristic for successful inclusion, through the demonstration of sporting abilities (4). The term inclusion often refers to a space in which individuals with disabilities are included (5). However, the term remains largely vague (6) and focused on education (7). In school sport research, one approach characterizing inclusion is through one's subjective experience (6, 8). According to Haegele and Maher (9, p. 390), subjective experiences are “dynamic, spatial, and in flux”. Although these authors primarily discuss inclusion as an intersubjective phenomenon, we apply the notion of dynamic and evolving experiences. As such, inclusion is not an absolute state that can be replicated in different spaces. In spaces designated as inclusive, feelings of inclusion may or may not occur depending on context and subjective experience (5, 10). In this research, we seek to understand inclusion as a subjective experience which complements the structural and institutional level of inclusion, in line with the United Nations Convention on the Rights of Persons with Disabilities (CRPD) (11). This level is expanded to understand how structural inclusion can be experienced subjectively through the perspectives of individuals with disabilities (12).

In this research, we seek to understand inclusion as a subjective experience. While our analytical focus lies on individual perceptions, we understand these experiences as socially situated and relational. Following Haegele and Maher (9), we acknowledge that subjective experiences are shaped through intersubjective processes—e.g., interactions with others or power relations. This individual level complements the structural level of inclusion as the systemic conditions enabling equal participation. By focusing on the subjective level, we aim to explore how structural inclusion is experienced by individuals with disabilities—identifying both supportive and limiting factors within inclusive environments.

Despite the testimony of the positive benefits of inclusion of sport by national and international sports organisations, individuals with visual impairment (VI) demonstrate a lower level of sport participation than their non-disabled peers (13). Moreover, they encounter specific barriers that hinder their participation in sport (14, 15). The reduced physical activity of individuals with VI in comparison to their sighted peers is attributed to a combination of factors (16). Largely, it turns out that barriers are often associated with “an inaccessible environment that is generally designed with only those who most closely embody normative values in mind” (17, p. 212). A lack of accessibility can be interpreted as an expression of ableism, a concept derived from disability studies that can be understood as an analytical tool.

Ableism refers to the process of both appreciation and devaluation of certain abilities. While abilities are viewed in an individualised way, the evaluation of abilities is often associated with the upvaluation and devaluation of entire groups of individuals (18, 19). As Nario-Redmond (20, p. 6) points out, “Ableism can operate at multiple levels affecting personal self-perceptions, interpersonal interactions, and intergroup relations”. Consequently, ableism can have an impact on the sporting participation of individuals with disabilities (17, 21), also among individuals with VI (14, 22). Challenges of this nature can be observed in a variety of sports, including skiing. Such challenges may be encountered at the organisational, physical and social levels, and are often faced by individuals living with disabilities (23).

The history of para-skiing dates from the Second World War where veteran amputees revived skiing in Germany and Austria. These were individuals experienced in skiing who developed methods to resume and continue skiing. In 1953, the first international competitions were held in Bavaria, Germany, but no blind skiers participated. The skiing movement developed rapidly and culminated in the first Paralympic Winter Games in 1976 (24, 25). Three starting categories are included at the Paralympic Winter Games: standing, sitting and since 1998 visually impaired. In para-skiing for athletes with VI, there are different categories for women and men: alpine skiing, cross-country skiing, biathlon (26). Skiers with VI usually ski with a sighted companion (guide) who provides perceptual and orientation aids (27). The organisation of guiding is subject to different guidelines, depending on whether the sport is recreational or competitive.

In addition to a sporting activity, skiing is seen as a meaningful leisure experience that focuses on health and social experiences (27–29). Winter sports have been shown to offer inclusive potential through their various opportunities for participation in the context of private excursions, club sports or commercial providers, as well as through a “high social and communicative density” (27, p. 417).

Despite the potential for inclusivity in skiing, the participation of individuals with VI remains under-researched. Current studies focus on the classification system in Paralympic sport (30, 31) and injury prevention (32). Mavritsakis et al. (33) investigated the factors that impact participation in adaptive snow sports for persons with disabilities, concluding that snow sports can provide opportunities for the acquisition of new skills and socialisation.

However, inclusion as a subjective experience for individuals with VI in skiing and the skills learned and integrated into their everyday lives is questionable. Ableism is understood as both a theory and a research perspective to tap into explicit and implicit potentials for exclusion from the perspective of individuals with disabilities (14). We chose the ableism-critical perspective to identify ability assumptions towards individuals with VI and to recognize the influence of abilities from the perspective of individuals with VI on their everyday activities. In this study, abilities are understood as subjective and socially constructed characteristics, whose value and meaning are shaped in the context of and in relation to disability (18). Ableism is complex and is interpreted in different ways. In our work, we view ableism as “those social, socio-technical and technical processes that attribute abilities and talents to individuals, groups or things, whether in an appreciative or devaluing manner” (18).

Using an ableism-critical and interpretivist perspective (34), this study explored the perceptions of adult skiers with VI regarding their own inclusion with a focus on abilities and the impact of alpine skiing on the experience of inclusion. Interviews were conducted, and an ableist-critical lens was used to identify implicit exclusive potentials.

## 2 Materials and methods

This study follows a qualitative, interpretivist epistemology and a constructivist ontology. We assume that experiences of inclusion are socially constructed and understood through the subjective meanings attributed by individuals with VI. Reality is multiple and constructed through social interactions and individual experiences (34). Our aim is therefore not to find objective truths, but to understand how individuals with VI make sense of their experiences of inclusion in specific contexts. In doing so, we are interested in the influence of sporting abilities. The open conceptualization of abilities allowed participants to express their own interpretations of ability without being confined to predefined categories. This is in line with our qualitative design and the use of episodic interviews to capture subjective meaning. It also shaped the interpretation of the findings.

This study is part of a broader research initiative on abilities and inclusion, and it aims to address questions that emerged from a study with blind tennis players (35). The central research question of this study is: How do abilities inform the experience of inclusion for individuals with VI? In addition, the study examines the following subquestions: (a) What experiences do alpine skiers with VI have in conversations about skiing with sighted individuals? (b) What skills do skiers with VI take away from participating in alpine skiing? (c) Do alpine skiers with VI experience their sport as inclusive?

### 2.1 Sample and data collection

To achieve this, episodic interviews (36) were conducted. The episodic interview makes it possible to access “knowledge, experiences and changes from the perspective of the interviewees” (36, p. 278). Episodic interviews are designed to access both episodic (situation-specific) and semantic (generalized) knowledge from participants. The interviewees are free to decide which experiences they wish to report. This creates different perspectives on how individuals subjectively interpret their experiences. In this way, the skiers' perspectives on the role of abilities in inclusive processes are made visible.

The first recruitment of participants took place through a trainer during a training course on “skiing with handicap”. No strict inclusion or exclusion criteria were applied during sampling; the main objective was to recruit individuals with VI who had personal experience in alpine skiing, regardless of their performance level. Interested persons contacted the second author on the basis of the study information provided. Further two contacts were found via the interviewees. Six individuals (five women, one man; average age 35) from six different German federal states volunteered to participate in the interviews. The participants provided electronic informed consent. The interviewees were both amateur and competitive athletes. Four interviewees had a competitive sports background. Of the four interviewees, two were no longer active in competitive sports at the time of the interview but were still skiing. All interviewees were experienced skiers, each had accumulated a substantial number of hours on skis during the years. Supplementary Table S1 in the appendix summarizes further information on the interviewees.

Interviews were conducted in German via Zoom in November 2024 by the first and second author and audio was digitally recorded after obtaining the participants' consent. The interviews lasted between 30 and 64 min (average time 43 min). The questions were asked in accordance with the established guidelines. The semi-structured interview guide based on the conceptual framework of the study was initially developed by the first author and subsequently revised in collaboration with the second author. The final version was the result of joint discussion and agreement between both authors. The interview guide contains these sample questions, among others:

• Can you describe situations in your everyday life where you apply skills you acquired through skiing?
• Thinking back to the time before you started skiing—how has skiing changed you as a person?

The sequence of questions was maintained, however, there was flexibility in the form, with questions excluded, rephrased or expanded upon as the interview progressed (36). All interviews were conducted and analyzed in German. After the interviews, all audio files were fully transcribed using the transcription software *sonixx*. All interviewees were assigned pseudonyms. The data was anonymized after transcription (e. g. cities, clubs, names). Selected sample questions and quotes were translated into English by the first author for the purpose of publication, with careful attention preserving the original meaning and context.

### 2.2 Data analysis

The data were analyzed using a content-structuring qualitative content analysis (37), to identify key themes and patterns relevant to the research question. The method of analysis is considered appropriate for episodic interviews (37) and has been used repeatedly in similar thematic contexts (8). The coding was carried out in a deductive-inductive hybrid form (37). Categorization was based on the approach of first defining *a priori* categories according to the interview guide. The material was initially coded deductively to obtain a structure. *a priori* categories *(Para)Skiing*, *Abilities*, and *Inclusion* functioned as a structuring framework and were further developed and differentiated in the analysis process. The three main categories were retained in the analysis process, whereby the category *(Para)Skiing* was renamed *Skiing* in line with the interviewees' use of the term. Subsequently, these categories were expanded to include subcategories in a multi-step inductive process.

**Figure 1:**
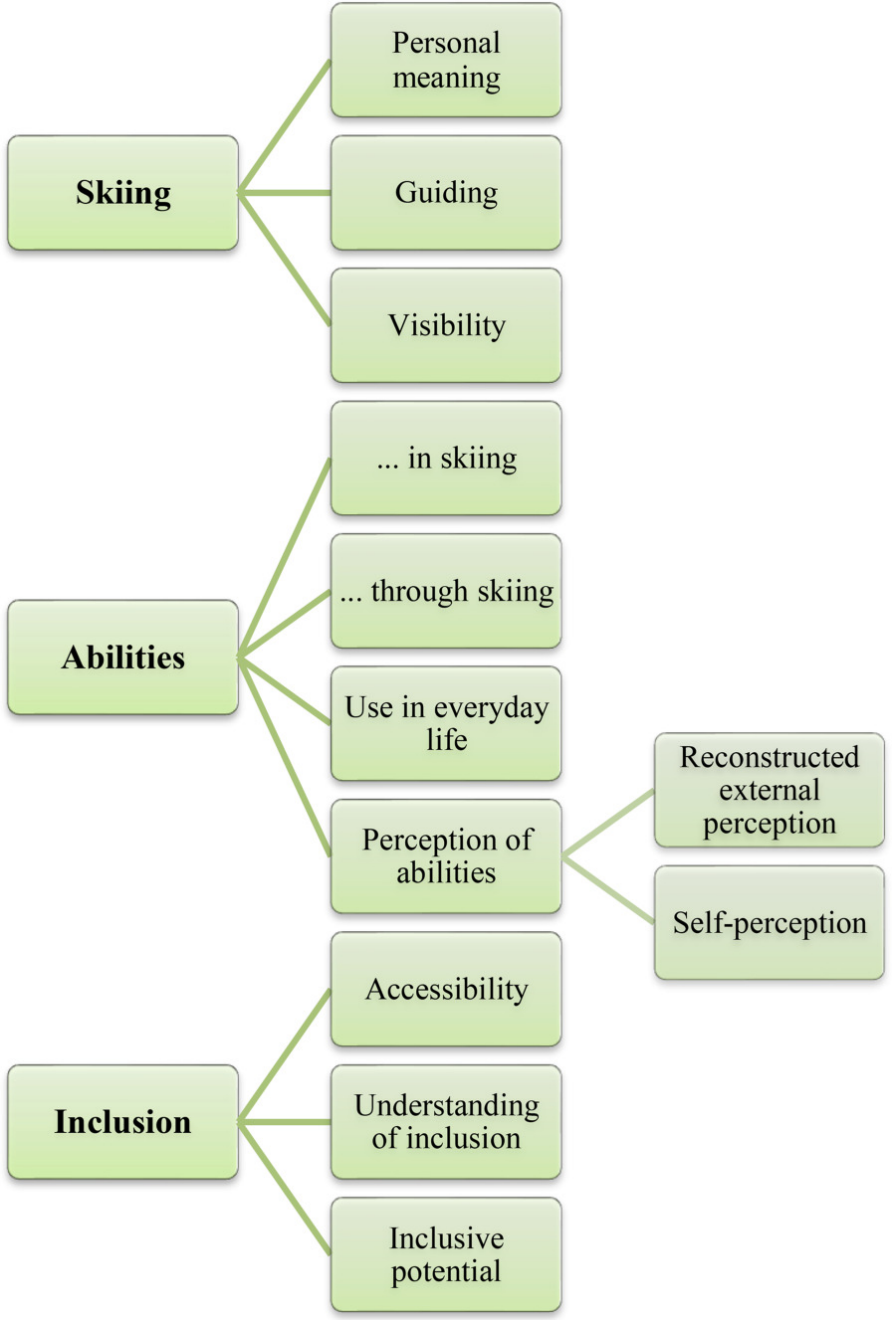
Coding system.

In the *Inclusion* category, the subcategories *Understanding of inclusion*, *Inclusive potential of skiing*, and *Accessibility* were formed. Following the initial coding process and the inductive determination of the subcategories, thematic overlaps were identified in the *Skiing* and *Abilities* categories. With the objective of addressing the research question—How do abilities inform the experience of inclusion for individuals with VI?—the subcategories were systematized and defined. In a subsequent coding process, the differentiated category system was applied to the six interviews, resulting in the combination of two subcategories: *Abilities for guiding* was subsumed under the subcategory *Abilities in skiing*, and the subcategory *Perception of abilities* was further differentiated into *Reconstructed external perception* and *Self-perception*. The complete and final category system (see Figure 1) was created by the first author and reviewed independently by the second.

In the final step of the analysis, the subcategories were not only grouped, but analytically synthesized across the cases to identify recurring patterns and shared meanings. Common experiences and underlying structures in the interviewees' narratives could thus be abstracted and thematically categorized. The interpretation was supported by case summaries, comparisons between the interviews and discussions between the first and second author. While participants at times reflected on how others may perceive them, the analysis focuses on the subjective experiences and interpretations of the interviewees. Thus, all findings are based on participants' individual reconstructions rather than on intersubjectively negotiated meanings.

A synthesis of the interview results revealed four key themes, which are presented in Chapter 3 Findings.

### 2.3 Trustworthiness

We have considered aspects of trustworthiness in the different phases of the research process (38). All authors have experience in dealing with individuals with VI through professional and private contacts. The first author works as a physical education teacher with children and adolescents with VI and also works in teacher education. The second author is a qualified ski instructor and works with children and adolescents with VI. Prior to the interviews, the first author was not familiar with the interviewees, while the second author had a previous academic connection with one participant. We acknowledge that this background may influence the research process and the interpretation of data. To enhance reflexivity and confirmability, we made our positionalities transparent and reflected on our roles throughout the process.

To support the rigor and quality of the study, we used various strategies. To support the internal quality of the study and to establish credibility, dependability and comprehensibility (37), the methodological procedure was set out in the methods section and additional material was provided in the form of figures and tables. To enhance authenticity of the study, we gave background information on the authors in relation to the study. Moreover, we have included a large number of quotes from the interviewees and aimed to reflect their language where appropriate. For example, identity-based language was frequently used by participants. To reflect the linguistic tone of the interviews and remain close to participants' terminology, we adopted the term “sighted persons” in the manuscript. At the same time, we consistently applied person-first language for individuals with visual impairments. To assess generalizability, Supplementary Table S1 provides additional information on the interviewees in order to assess the transferability of the data to other contexts. Further limitations are discussed in detail in the Limitation section.

## 3 Findings

The results of this study provide insights into the subjective experiences of athletes with VI in alpine skiing, with a focus on their perceptions of inclusion and abilities. A detailed analysis of the interviews revealed that the six interviewees exhibited notable parallels in their experiences, challenges, and perspectives on alpine skiing despite their varied backgrounds and life paths. The focus of the interviewees was primarily on skiing with social aspects being discussed marginally. The primary focus of the analysis was the *Abilities* category, which has a close connection to the subcategories *Visibility*, *Inclusive potential* and *Accessibility.* These subcategories were also included in the analysis.

A synthesis of the interview results revealed four key themes: skiing as a booster for self-confidence; skiing as proof of expertise and competence for oneself and others; skiing under the radar; skiing as a pathway to accessibility and inclusion. The findings are presented in detail below.

### 3.1 Skiing as a booster for self-confidence

All interviewees emphasized that skiing enabled them to enhance their self-confidence. Through skiing, they developed personal competencies such as adaptability, self-confidence, organizational skills, and problem-solving strategies in their daily lives. For instance, Lara and Lilli described how they applied their gained self-confidence in everyday life when dealing with their visual impairment:

“But skiing was just a big part of me just dealing with my visual impairment really confidently and just openly saying what I need.” (Lara)

“And that gave me a bit of courage to think that I can and should try everything, even if you think in advance: can it work?” (Lilli)

Karin and Peter emphasized the flexibility they had gained in dealing with challenges or unforeseen circumstances:

“Because you are always in the situation, which of course happens to you in everyday life, that something goes completely differently than you imagined and then you have to see how you can get on.” (Karin)

“So I'd say the phrase would be: “There's always another way’. If one thing doesn't work, then we just have to try something else.” (Peter)

Parallels can be drawn to the research of Conroy (39) on recreational ski programs for children, in which skiing was found to contribute to building trust and self-esteem, developing self-confidence, and thereby overcoming everyday challenges. The interviewees in our study identified not only personal competencies but also additional skills they had acquired or further developed through skiing. They described an improved body awareness and enhanced coordination skills because of skiing. These terms were used by the interviewees in various contexts and were not always clearly distinguishable in meaning. Many interviewees perceived their improved body awareness as an advantage in practicing other sports, such as fitness, climbing, or cycling. Additionally, the motor and sensory skills they had acquired played a significant role in their orientation and mobility in everyday life:

“It's very good training for balance. It may be a bit high, but it's definitely also a bit of fall prevention.” (Karin)

“I'm now quicker on my feet, which also helps me in everyday life. I simply have more overall tension. I'm more stable. I have a better sense of balance. Even at a standstill.” (Peter)

“[..] that I walked through the city in a completely different way, that I, um, that my physical body perception changed a lot and I had much less orientation before and that gave me a lot.” (Nora)

Against the backdrop of altered orientation in snow and ice (40) improved skills through skiing can contribute to safer movement during the winter months (27). The significance of skiing fostering orientation and mobility has been demonstrated in studies conducted with children with VI or blindness (39). Other sporting activities have also been shown to contribute to the development of orientation and mobility skills, as evidenced by research conducted by Oldörp et al. (35) in adults.

In addition to sports motor skills and ski-specific techniques, the interviewees highlighted social and personal skills (e.g., organizational skills, reliability, teamwork, communication, courage, and fearlessness) as well as cognitive and sensory skills (e.g., mindfulness, cognitive flexibility, concentration, body awareness, visual and auditory perception) as the foundation for alpine skiing. The interviewees also identified specific skills for skiing with visual impairment or blindness:

“So right now, we also have to speak, listen and react at the same time and then we have to implement this in our bodies.” (Nora)

“[..] and then I can also combine the visual with what I can hear, so to speak. I get the commands acoustically and I can also see my guide quite well.” (Helena)

Guiding played a particularly significant role for all interviewees, with all six athletes describing specific skills required from both the skier and the guide: teamwork, communication skills, trust, and adaptability.

“The ability to build trust and, when you have it, to rely uncompromisingly on the command.” (Peter)

“And I believe that I can ride with almost anyone, because I can explain what I need in relatively simple terms.” (Nora)

Similar parallels can be drawn with the studies by Hiemstra and Rana (10) as well as Ball et al. (16) on runners with VI, and Macpherson (22) on blind hikers with their guides. In their studies, trust and communication were identified as key factors for a successful running or hiking experience.

### 3.2 Skiing as proof of expertise and competence for oneself and others

The categories of *Reconstructed external perception* and *Self-perception* distinguish between “the experience of abilities and the attribution of these abilities” (18). This theme will be explored in more depth in the following subthemes.

#### 3.2.1 Reconstructed external perception of abilities

The *Reconstructed external perception* of abilities is based on the interviewees' subjective descriptions of how they think they are perceived by sighted individuals in their sport or everyday life. This shows an interaction between the perception of one's own ability and the assumed assessments by others.

“I want to be perceived as a human being and not as the blind one.” (Peter)

However, these assessments are not always based on direct feedback from sighted individuals, but on the experiences and interpretations of the interviewees. In some cases, interviewees reported being ascribed or denied abilities; however, these attributions were often made based on their own perceptions and not necessarily on the basis of explicit statements or assessments by sighted individuals. In this study, the category *Reconstructed external perception* does not refer to an objectively collected perspective, but rather a perception reconstructed from the interviewees' stories. This reconstruction was achieved through an ableism-critical lens, and it does not necessarily reflect explicit statements by the interviewees. Rather, it reflects the social interpretation patterns that are recognizable in their descriptions.

In both skiing and in their everyday interactions at work, university or in their leisure time, the interviewees had experiences that they interpreted as reactions from others to their abilities. In the interviews, they described situations, experiences and conversations with sighted individuals. According to the interviewees' accounts, they experienced a range of reactions that were interpreted in the coding process as (a) admiration and appreciation and (b) underestimation and surprise.

(a) Admiration and Appreciation

The interviewees reported positive feedback received for their achievements in skiing or other areas of their lives.

“And um, yes, then I remember a situation where I did a parallel slalom against a non-disabled person and um, yes, the times were really close. [..] And then you get to the finish and you also get the appreciation from the other person.” (Helena)

“That's what the coach [from one sport] always says, too, that it's really amazing how my body feeling is compared to the other athletes [..].” (Lara)

There are notable parallels with the supercrip motif, which is characterized by a specific perspective on individuals with disabilities, emphasizing their abilities and achievements in a manner that glorifies and idealizes their capabilities. This viewpoint is problematic, as it presents a stereotypical and distorted depiction of the abilities of individuals with disabilities (41). An opposite reaction was shown in subtheme (b) underestimation and surprise.

(b) Underestimation and Surprise

In contrast to admiration, the interviewees also described situations in which their abilities were doubted or met with disbelief. The interviewees shared how surprised observers were by their competence and performance on skis. For example, Helena recalled feedback she received after a competition:

“Wow, that run went really well for you. And your times are really close to non-disabled times, basically.” (Helena)

This demonstrates that certain normative concepts of skiing are not transferable to the ideas of skiing held by individuals with VI. This is also evident in the following question that was posed to Nora:

“If we can drive fast and things like that.” (Nora)

Ableist assumptions about the abilities of individuals with VI can lead to the underestimation of their capabilities. Closely linked to this are doubts about the abilities of individuals with VI, such as concerns regarding potential risks or dangers:

“So that's the classic question: “How do you even see the slope?’ or “Aren't you overlooking people?’ I think that's also a bit of a fear with other people. Especially if they are skiers, as if I could drive over them.” (Lilli)

Karin expressed similar thoughts about her experiences. She emphasized afterward that individuals with VI are not only capable of skiing, but also possess knowledge of implicit rules:

“We are not a risk to the environment or to ourselves, we have learned it. We have learned the sport and we want to do the sport. And we know that we have to stick to certain rules.” (Karin)

Comparable experiences were also reported by interviewees in everyday situations:

“Then they want to practically shove a chair under my butt. Or they give me a fork and say: “This is a fork.’ Which makes me think: Hello?!” (Peter)

In the situation outlined by Peter, individuals with VI experienced a denial of certain abilities, which can be attributed to the social construct of disability and its subsequent influence on perceived visibility. “It is assumed that abilities are not something that can be described ontologically, but rather a *relational* [italics in original, FO] variable that is tied to the social position of the observer and thus ideologically shaped.” (18). Peter described a situation in which his skiing abilities served as a primary source of information about his identity, leading to a misperception among sighted individuals regarding his disability status:

“And then we drove, swung off at the end and then I was standing next to someone who looked at me, looked at my glasses and said: “How can you see with these?’ And then I just pointed to my jersey and he just collapsed.” (Peter)

Similarly, the interviewees in the study by Ball and Haegele (14) reported that individuals did not believe that they were visually impaired.

#### 3.2.2 Self-perception of one's own abilities

*Self-perception* refers to how the interviewees perceived their own abilities and how this perception influenced their self-image and actions. The self-perception of the interviewed skiers is characterized by pride, self-confidence, and an active approach to their disability. Skiing is seen by the interviewees to strengthen self-confidence and experience themselves as capable. Strong interactions exist between the categories of *Reconstructed external perception* and *self-perception* of abilities. The enhancement of self-confidence through skiing is described by all six interviewees in different contexts. The perception of their own abilities and the associated achievements in skiing emerge as crucial elements in the development of self-confidence and pride for all six interviewees.

“I would say it boosted my self-confidence because I realized that I'm more athletic now and have some skills, I would say, and can keep up quite well with others who don't have a disability. And that has really boosted my self-confidence.” (Helena)

“Well, I was self-confident before, I wasn't hiding, but I just realized: Yes, I can do something here that others can't, that others also think is kind of awesome. Um I'm certainly a bit proud of myself as a result.” (Peter)

This issue is also closely related to the management of one's own visual impairment in both sporting activities and daily life. It encompasses more than simply demonstrating competence to others; it also involves navigating self-imposed limits, as Lilli and Karin articulated:

“And I think it [skiing] has made me more self-confident, because I know that a visual impairment doesn't just have to mean limitations, but that somehow everything is possible.” (Lilli)

“For the disability itself, I think I'm just incredibly happy that it works, and I think it's nice that there are ways and means of overcoming some of your own prejudices [..].” (Karin)

It is possible that Karin's “own prejudices” may be attributed to internalized ableism (14). A similar interpretation can be applied to Lara's statement:

“You often start to say, you're often taught from the outside, “Okay, that's not possible because of the disability’ [..].” (Lara)

These internalized prejudices can have a negative impact on self-perception and affect other areas, so that individuals do not feel confident doing certain sports or other activities in everyday life (17). Moreover, these prejudices can result in feelings of exclusion from inclusive environments, potentially due to perceived inadequacies caused by ascribed abilities (42).

Skiing seems to take on a special significance when abilities are negotiated with oneself and with sighted individuals.

“I think that's always an ace up my sleeve [..] if I feel a bit smaller because of my visual impairment, I can still say—“I'm skiing, maybe you're not’. That gives me a lot, because I know that's an ability you can be proud of and maybe even more so with my visual acuity. So yes, it does give me a little something that I can do that.” (Lilli)

“When I talk about my visual impairment, because then it's not directly perceived as a weakness, but okay, she is still strong. Or in general, when I talk about everything else I do. So that really strengthens me.” (Lara)

Through skiing, the interviewees experienced a shift in how they were perceived by others. As May-West et al. stated (29, p. 362), having “an activity that any sighted person has the opportunity to do, is something that builds confidence and helps to challenge the perceptions of others to see those with significant visual impairment as equals”.

### 3.3 Skiing under the radar

All six interviewees reported on conversations with different individuals (colleagues, friends, fellow students) in different contexts (on the ski slopes, in everyday life) about skiing. The conversations with others revealed that para-skiing is often an unknown sport:

“Sometimes I get the feeling that people think: “Yeah, she's taking the piss, as if she's alpine skiing’. And then they look on the internet and see, yeah okay, she really does that.” (Nora)

“I think it's more of a brief shock or surprise. But yes, it is. You get asked more because it's something special.” (Lilli)

In their conversations about skiing, the interviewees expressed a lack of knowledge on the sport, but also a profound level of interest in learning more about the sport. On occasion, these conversations also revealed a high degree of appreciation for skiing in conversations:

“Yes, I also sometimes find it strange when someone celebrates skiing as incredible and I think: yes, basically I do the same as you, I just ski down there.” (Peter)

The athletes used the conversations to raise awareness of para-skiing and break down prejudices.

“Yes, mostly, because some of them have never heard of it or never seen it or something. [..] So, for example, they grew up somewhere in [town] or whatever and were skiing in their ski area and saw people with one jersey on, skiing behind each other and shouting at each other. Then they usually couldn't classify it beforehand and thought yes, they're having fun and then, when they talked to me and we talked about alpine para-skiing, they could just classify it [..].” (Helena)

Many of these conversations took place through an equal professional exchange (Karin) or to establish contact with other individuals (Helena).

“Now the season is starting again, at the moment it's all about gaining experience with protectors and recommendations and collecting a bit of information about what's available.” (Karin)

“So, it's just a normal conversation at the same level with people who don't have a disability. Especially at university now, [..].” (Helena)

The limited visibility of the sport was identified as a significant concern and could be attributed to the limited awareness among sighted individuals, and secondly, to the media's lack of interest, and the limited involvement of spectators. However, amidst these concerns, some interviewees also noted positive developments in visibility, though they did not provide specific examples.

“But apart from that, at the races it's really, really sad how few spectators there are. I also raced in the World Cup and even in the World Cup there's simply no one there. In fact, sometimes nobody. At most there are parents who come by, acquaintances who really know an athlete.” (Lara)

“And I think it's a shame that there is this threefold division [Olympics, Paralympics, Special Olympics]. And on the other hand, yes, visibility has increased significantly in recent years and that's important.” (Karin)

The skiers expressed their appreciation for sporting events held alongside non-disabled athletes, citing the increased presence of spectators and the opportunity to compete against a wider range of skiers as key benefits.

“But this ski league is simply with non-disabled athletes. And um, yes, that's actually the most fun for me, to be honest. Because there are a lot more people taking part and the one or other spectator sometimes.” (Helena)

Separate structures not only prevent joint contacts, but also minimize visibility. Karin points out that without visibility, the achievements of individuals with VI cannot be adequately highlighted:

“[..] and the hope would of course be that it would then have a further impact on the general labor market and that you simply see that people have more potential than you think they have at first glance.” (Karin)

There are parallels here with the aims of the Special Olympics, which utilize sport as a means to draw attention to the potential of individuals with disabilities (4).

### 3.4 Skiing as a pathway to accessibility and inclusion

The skills and competencies learned and improved through skiing were used by the interviewees in their everyday lives, e.g., in other sports or in orientation and mobility, as described above, which gave them greater access to sports and exercise culture. The skills learned through skiing also played an important role in relation to the lack of accessibility in the interviewees' everyday lives. The interviewees experienced a lack of accessibility for individuals with VI primarily in the context of work and university, as well as in their school life or previous sporting activities:

“And then handball is still more about competition in the junior phase. Yes, and then it just wasn't so much fun for me anymore, because before that, for example, people were always calling my name or something so that I could pay attention and concentrate to catch the ball and then they weren't allowed to do that afterwards because it gave the opponent an advantage and stuff like that.” (Lara)

“I would very much like to see digital accessibility considered in all programs from the beginning and not to be presented with a program afterwards: Yes, we've decided on this, it's convenient for everyone. Oh, you can't use it?” (Karin)

“Yes, that all the documents are accessible to me and that's rarely the case. So yes, integration. Yes, I would say it's integration, because I'm allowed to study, but it's not inclusion, because I have to run to the front every time. There are-, I think now there were maybe two profs where I didn't have any problems.” (Lara)

Moreover, a significant number of interviewees identified a general lack of accessibility in their daily lives.

“Well, it would be nice to get to a point where you no longer have to request your rights [..].” (Karin)

“At the climbing center, for example. I don't know if you've ever been there, but the numbers of the boulders are up there. [..] Yes, and then I always walk around there with my mobile phone and take photos of the routes and zoom in on the numbers. Little things like that. That's the simple solution in itself, but it's just something that happens so often.” (Lara)

The interviewees reported how they used the abilities they had acquired through skiing in everyday life to highlight exclusionary issues in their studies or work. For example, Helena points out a lack of accessible work documents to their teachers:

“That all materials are uploaded to the learning platform we use from the very beginning. That there is simply an extra folder. Accessible slides and a folder with normal slides, let's put it that way, and that you don't have to ask for it, it's just there. That would be my ideal, my ideal vision.” (Helena)

They also used their experience and abilities to motivate their peers with VI:

“I'd just say do what you feel like doing and don't let the fact that someone says it won't work stop you. Go for it.” (Lara)

Standing up for their own interests sometimes required courage and tenacity from the interviewees. For many, the self-confidence gained through skiing proved to be the main driving force:

“[..] and in regular school I wouldn't have had the courage to go to the front all the time and say yes, anyway, you have to do this on my laptop now.” (Lara)

“That some of my work is not accessible. And I don't think that ten years ago I would have been so insistent and wouldn't keep asking and asking and asking that it works and that I can do my work without obstacles.” (Nora)

Accessibility was an important topic in the interviewees' everyday lives, and particularly in skiing. The interviewees reported no barriers in relation to skiing and generally saw skiing as inclusive. All six interviewees viewed it as positive “that skiing is actually really good even with a visual impairment if you have a guide” (Helena) and that shared experiences are constructed through “the same passion” (Peter). May-West et al. (29) also arrived at the conclusion that skiing can be regarded as inclusive due to the fact that the sport can be adapted to the differing needs of athletes. In the present study, the methodological adaptations and the practice of skiing with guides can also be regarded as an obstacle to inclusion, as evidenced by the interviewees' narratives, insofar as these symbolize deviations from the skiing of sighted individuals and label those individuals as visually impaired:

“Because it doesn't matter whether you're on the slopes with a speaker, with a guide or whether you're on your own with a white cane. You never get lost in the crowd. [..] People are watching anyway, whether I'm on the slope or not.” (Karin)

A lack of guides, on the other hand, can be seen as an obstacle to participation and inclusion:

“And the first year I think I only went on two courses because I was looking for a guide and the structures weren't there yet. I had to find a guide myself.” (Lara)

As Macpherson (22) and Ball et al. (16) also found, participants in their respective studies, identified the lack of availability of guides as a significant barrier to participation in hiking or running. The data in our study revealed that all interviewees were guided by family members at the beginning of their skiing careers—a practice which continues. Guide changes occurred when family members were unavailable or when the skiing skills between the skier and guide were great. As posited by Ball et al. (16) the investment of time required to find a new guide, and to establish a shared understanding of skiing was also a barrier. Another salient barrier on the slopes is the presence of other skiers who ski between the guide and the skier, despite being specially marked:

“We also have the problem that if, for whatever reason, the distance between us becomes too big or it gets too busy, then people drive between us.” (Peter)

The lack of joint events for individuals with and without disabilities was also an obstacle to inclusion, as described above in relation to competitions. This also applies to the organization of recreational skiing trips.

“But that's something that is actually the responsibility of the organizers. Because if I organize it from the beginning so that the sighted and the blind are in two different accommodations, then it's no longer inclusive.” (Karin)

Nevertheless, the interviewees' conversations in their private lives also lead to contacts through skiing, e.g., at university:

“Especially at university now, because [the city] is a place where people ski, and that's why a lot of people ski at university and then you talk about it and most of them think it's really cool and then you go skiing together and can connect easily, I think.” (Helena)

To summarise, it can be posited that the interviewees developed a variety of sporting, social and cognitive skills through skiing, which play a central role for them both in their everyday lives including their sporting experiences. These findings underscore the role of abilities in promoting inclusive processes and reflecting on the implications for practice. The following section is dedicated to the interpretation of the results in relation to our research questions and their placement in the existing research context.

## 4 Discussion

In consideration of the research question—How do abilities inform the experience of inclusion for individuals with VI?—it is evident that abilities played a significant role in the interviewees’ narratives. On the one hand, the interviewees used their abilities in everyday life and to manage a lack of accessibility. On the other hand, the interviewees' narratives can be interpreted from an ableist perspective implying certain assumptions about the abilities of individuals with VI. There is a need for a critical reflection on ableist constructions of ability, derived from the interviewees' narratives and based on their subjective interpretations. These perceptions are often shaped by societal notions of individuals who are blind, not grounded in direct knowledge but rather in prejudices and assumptions. These prejudices are influenced by “purely visual perception and interpretation patterns” (43, p. 212). Communication styles can reinforce existing prejudices and conceptions of disability, but conscious communicative processes can also challenge and reshape conceptions of disability (20).

However, it is important to acknowledge that all participants were highly experienced and committed to skiing. As such, the positive effects described—particularly the boost in self-confidence—reflected the perspectives of those who are and remained engaged in this sport. The experiences of individuals who may have discontinued skiing due to challenges or exclusion were not captured in this study and warrant further exploration. This limitation also highlights the need for further research into the inclusion experiences of those who have faced difficulties accessing or continuing participation in alpine skiing.

With regard to subquestion (a)—What experiences do alpine skiers with VI have in conversations about skiing with sighted individuals?—it can be stated that the athletes used the opportunity in the conversations to make contacts and provide information about the sport. This can have the effect of changing external perceptions and increase visibility, especially if the conversations are mutually respectful and beneficial (20). The interviewees' experiences in the conversations are often positive but tend to be accompanied by admiration and a lack of knowledge about para-skiing. According to Nario-Redmond (20), frequently exaggerated positive assessments of the actions of individuals with disabilities arise from both benevolent attitudes and low expectations. The knowledge about alpine skiing consists of alpine skiing of sighted individuals, based “on normative, ableist structures, which set this specific sports context as the norm or “ideal’” (10, p. 4). Interactions between individuals with and without VI can challenge certain everyday practices (43). In this context, skiing serves as a mediator, as the interviewees shared their skiing experiences with others, demonstrating that individuals with VI can ski and engage in similar recreational activities as sighted individuals. In doing so, the skiers challenged ableist perspectives and contributed to both the normalization of the sport and the broader recognition of individuals with VI. Similar findings were reported by cross-country skiers with VI by May-West et al. (29).

However, the practice of using a sport as a means of demonstrating ability should be critically examined, as access to sports and physical activity for individuals with VI remains limited by various barriers (14, 15, 44–46) preventing some individuals from engaging in sports. Moreover, this perspective excludes individuals who, for various reasons, are unable to present themselves as capable (47). The notion of demonstrating abilities to replace the category of disability has been critically assessed by Oldörp et al. (48). Further research is needed, particularly involving individuals with disabilities (12).

Regarding subquestion (b)—What skills do skiers with VI take away from participating in alpine skiing?—two key areas can be identified: motor skills for sports and mobility, as well as personal competencies for everyday life. The interviewees increasingly advocated for their needs and rights, citing skiing as a source of their increased self-confidence. The significance of self-advocacy as an ability was confirmed in the research conducted by Ball et al. (16) and Rich et al. (45) with runners and rowers with VI. The ability to advocate for one's own interests is recognized in both studies as a crucial factor for participation in sports and competitions, particularly when inclusive structures are lacking or access to opportunities is restricted. Our study demonstrates that skiers primarily engaged in self-advocacy in the areas of work and education. Lieberman and Childs (49) advocate for teaching self-advocacy skills to children and adolescents to enhance their participation in school and recreational sports. However, this perspective appears problematic, as successful inclusive processes should not depend solely on the individual and their abilities. Framing inclusion in this way risks neglecting the need for broader societal change.

Nevertheless, improving sports participation among children and adolescents is a crucial measure, as access to physical education is often limited (46, 50) and inactive students are more likely to become inactive adults (5). In addition to the physical barriers, ableist body narratives (51), standardized performance expectations (52), and teacher behavior (5, 53) contribute to negative experiences in physical education. The findings of Sträter and Stegemann's study (54) on guiding of skiers with VI in sport teacher education highlight the importance of addressing inclusion-specific topics within teacher education programs. Through alpine para-skiing, students developed a positive attitude toward a specific aspect of diversity. Further research should examine whether this effect can be replicated in other sports contexts and with a broader concept of inclusion. These findings align with the recommendations of Grenier and Giese (55), who argue that university-based physical education teacher training must incorporate critical perspectives on ableism to reflect on exclusionary mechanisms within school sports. This perspective should be extended to the training of coaches across all performance levels (56), instructors, and other professional groups to reduce disability-related biases (45, 57).

Dealing with the continued inaccessibility of sport opportunities and a lack of barrier-free environments can be exhausting. Coping strategies, personal competencies, or additional skills are necessary to address these challenges and maintain motivation for sports (14). Sports activities themselves (13, 58) or spending time in nature (59) are frequently used as coping strategies. “However, opportunities for these experiences, momentary or otherwise, were much harder to find due to the ableist conceptions of risk and vulnerability often encountered with sight impairment” (59, p. 318). This becomes particularly problematic when ableist conceptions and prejudices are internalized by individuals with VI. “Internalized ableism is defined as the process of projecting negative thoughts and feelings onto oneself based on societal stereotypes surrounding disability. In instances of internalized ableism, disabled people internalize society's beliefs that being disabled is an inferior form of being […]” (14, p. 2). Traces of internalized ableism were occasionally evident in the statements collected in our study which can influences competence (17). However, skiing helped the interviewees perceive themselves as competent and capable. Similar findings were observed in the study by Oldörp et al. (35), in which blind tennis players developed “competent identities” through their sport. Participation in sports thus emerges as a crucial factor, particularly given that individuals with VI engage in less physical activity than their sighted peers. The reasons for this lower level of physical activity are often linked to a lack of accessibility and internalized ableism. Diverse experiences of individuals with disabilities are essential for understanding and analyzing inclusion experiences and for drawing relevant conclusions for sports practice (10).

Regarding subquestion c)—Do alpine skiers with VI experience their sport as inclusive?—no definitive conclusions can be drawn. From the perspective of the interviewees, skiing is inclusive because it is accessible to individuals with VI, a finding supported by the study of May-West et al. (30). However, skiing is not perceived as inclusive when, as previously discussed, separate structures exist for athletes with and without disabilities in both competitive and recreational settings. Separate competitive structures were perceived by some athletes as non-inclusive and limiting the visibility of the sport. A lack of spectators and the sport's low public recognition were also reported as negative experiences among blind tennis players (35).

For individuals with disabilities, ski trips often require additional planning efforts to find accessible services and routes (e.g., transportation, accommodation, and local facilities) and to secure limited spots in advance (60). Guiding was generally viewed positively in our study, which may be attributed to the fact that all interviewees had extensive skiing experience and were well-connected within the community. However, for beginners and occasional skiers, the lack of available guides could represent a barrier to participation. That missing guides can hinder sports participation was demonstrated in the work of Ball et al. (16) and Macpherson (22).

The connection between (lack of) accessibility and feeling inclusion—the presence of feelings of appreciation and belonging from the perspective of people with disabilities—is an important factor when evaluating spaces designated as inclusive. As Maher et al. (61) demonstrate in their qualitative study, subjective feelings of inclusion are often closely linked to environmental and structural conditions in sport. Based on our data, it is not possible to make a clear judgment on this matter. Further research is necessary to investigate the relationships of feelings of inclusion through acceptance, belonging, and value.

## 5 Limitations

This study has several limitations that must be considered when interpreting the results. First, the small number of participants limits the generalizability of the findings. Additionally, the study focused exclusively on the sport of skiing and individuals with visual impairments or blindness, which narrows its scope. Furthermore, it is important to recognize that statements about sighted individuals should be understood as subjective reconstructions. Since the study examines subjective perspectives, its results cannot be generalized. Future research should include larger and more diverse samples, explore additional sports, and consider the perspectives of individuals with different types of disabilities. Nevertheless, certain theoretical patterns can be recognized that could be transferable. This applies particularly to patterns concerning the role of abilities for oneself and others, visibility and the negotiation of accessibility. This aligns with results from other studies on individuals with VI in sport (see among others 16, 44, 45).

The chosen methodological approach also has limitations. The interpretation of the data through qualitative content analysis following Kuckartz (37) is shaped by the researchers' perspectives and prior knowledge. This subjectivity is inherent to qualitative research. Reflexivity and discussion were used to ensure transparency and analytic rigor. A theory-driven categorization process can result in certain aspects of the data being emphasized while others are potentially overlooked (37). The methodological approach of episodic interviews also presents challenges, particularly when interviewees describe experiences they have not personally had but have heard about or assumed (36). Additionally, both competitive and recreational athletes were interviewed, which introduces differing perspectives on the sport and the broader sports system. This might influence the perspective on the inclusive potential of skiing.

Skiing is a seasonal sport, meaning that even for experienced skiers, the time spent on skis each year is often limited. The relatively short and sometimes irregular periods on skis can influence how skiing-related skills are reflected upon. All interviewees had extensive skiing experience and were well connected within the skiing community, even as recreational athletes. However, it remains unclear to what extent their experiences can be applied to beginners. A larger sample is needed to capture a broader range of experiences. Furthermore, the deeper relationship between skiers and their guides was not explored in detail, presenting an additional avenue for future research.

Another important factor is the financial cost associated with skiing, as expenses for equipment, lift passes, transportation, and accommodation must be covered (39). This financial barrier may mean that access to the sport is predominantly available to individuals with higher socioeconomic status. Additionally, socio-cultural and ethical aspects must be considered, as access to skiing is often shaped by family traditions, societal structures, and regional factors. In many countries, skiing is a sport with specific traditions and access structures that are not equally available to all social groups. These factors may have influenced our research findings and should be more thoroughly considered in future studies.

Despite these limitations, the study provides valuable insights into the subjective experiences of skiers with VI and highlights how specific sports can help foster inclusive potential and challenge ableist structures. In particular, the combination of individual perspectives with critical reflections on normative assumptions about abilities offers practical insights for the further development of inclusive recreational and sports opportunities.

## 6 Conclusion

Using an ableism-critical and interpretivist perspective, this study explored how sport-related abilities influence inclusive processes from the perspective of skiers with visual impairments. The findings highlight the significance of sports participation for individuals with disabilities, both in physical, psychological, and social terms. To ensure equal participation, it is essential to eliminate existing barriers while simultaneously providing a broad range of inclusive and peer-group-specific (29) sports opportunities. This enables individuals to choose an option that best suits their individual needs and preferences (7, 22). However, inclusion is not solely about the spatial and structural availability of such opportunities. It also requires raising awareness among sighted athletes, coaches, and other staff members to ensure the sustainable implementation of inclusive and barrier-free sports programs.

This study highlights the role of alpine skiing in fostering confidence, competence, and inclusion for individuals with VI. Participants challenged ableist assumptions but faced barriers such as limited visibility, segregated competition, and inadequate guiding support. To improve experiences of inclusion, awareness must be raised, accessibility enhanced, and integration into mainstream skiing promoted. Shared experiences between disabled and non-disabled athletes can bridge gaps and foster a more inclusive sports culture. Future research should explore broader contexts and diverse sports to deepen the understanding of inclusive practices. Ultimately, this study underscores that abilities should be seen as assets rather than limitations, advocating for inclusive approaches that ensure equal participation for individuals with disabilities in sports.

## Data Availability

The datasets presented in this article are not readily available because the dataset consists of qualitative interview transcripts that contain personal reflections of participants. Due to confidentiality agreements and ethical considerations, the full dataset cannot be shared to protect participant privacy. Requests to access the datasets should be directed to Felix Oldörp, oldoerpf@ph-heidelberg.de. Please note that the dataset itself is not available due to confidentiality restrictions.
