# Supplementary material for: The importance of abilities in inclusive experiences from the perspective of people with visual impairments: the example of alpine skiing
Source: Front Sports Act Living. 2025 May 30;7:1587603. doi: 10.3389/fspor.2025.1587603 (PMC12162934; doi:10.3389/fspor.2025.1587603)
Supplement: Supplementary file 1 [file Table1.docx]

Supplementary Material

Table 1 Characteristics of the respondents (on the date of the interview)

| **Name (gender)** | **Age** | **Profession** | **VI onset & course** | **Sport Class for Para Alpine Skiing^[[1]](#footnote-1)^** | **Skiing experience** | **Skiing days per season** | **Path to skiing** | **Competition Experience** | **Club** | **Other current sports** |
| --- | --- | --- | --- | --- | --- | --- | --- | --- | --- | --- |
| **Lilli (woman)** | 25 | student | congenital, stable | / | since age 16 | 1-15 (currently) | through family | no | no | hiking, fitness |
| **Peter (man)** | 59 | retired | congenital, progressive | AS1 | since age 20 | 30-40 (currently); 60-70 (previously) | / | in the past | yes | fitness, indoor cycling |
| **Helena (woman)** | 20 | student | acquired | AS3 | since age 4 / since 2020 competitive | 110-130 (currently) | through family | yes | yes | mountain biking, climbing |
| **Karin (woman)** | 51 | employed | congenital, progressive | AS2 | since age 3 | 20 (currently) | through family and club | no | yes | hiking, fitness, swimming |
| **Nora (woman)** | 33 | employed | acquired | AS2 | since age 3 | 120 (previously) | through family | in the past | yes | fitness, swimming, mountain biking, jogging, inline skating |
| **Lara (woman)** | 23 | student | congenital, progressive | AS2 | since age 3 / since 2018 competitive | 120 (currently) | through family | yes | yes | hiking, fitness, mountain biking, climbing |

1. The VI classification in para-alpine skiing is divided into four categories from AS1 (light perception without measurable visual acuity or no light perception) to AS4 (visual acuity LogMAR 0.6-0.9 or the binocular visual field is constricted to a diameter ≤ 70°). This classification is based on the classification for blind and visually impaired athletes (B1-B3) in the Paralympic sports. (59). [↑](#footnote-ref-1)
